# A three-protein biomarker panel assessed in diagnostic tissue predicts death from prostate cancer for men with localized disease

**DOI:** 10.1002/cam4.281

**Published:** 2014-06-07

**Authors:** Gianluca Severi, Liesel M FitzGerald, David C Muller, John Pedersen, Anthony Longano, Melissa C Southey, John L Hopper, Dallas R English, Graham G Giles, John Mills

**Affiliations:** 1 Cancer Epidemiology Centre, Cancer Council VictoriaMelbourne, Victoria, 3004, Australia; 2 Centre for Molecular, Environmental, Genetic and Analytic Epidemiology, School of Population Health, The University of MelbourneMelbourne, Victoria, 3010, Australia; 3 TissuPath Specialist PathologyMount Waverley, Victoria, 3149, Australia; 4 Faculty of Medicine, Monash UniversityVictoria, 3800, Australia; 5 Genetic Epidemiology Laboratory, Department of Pathology, The University of MelbourneMelbourne, Victoria, 3010, Australia; 6 Macfarlane Burnet Institute for Medical Research and Public HealthMelbourne, Victoria, 3004, Australia

**Keywords:** AZGP1, immunohistochemistry, MUC1, NKX3.1, p53, prognostic markers, prostate cancer-specific death

## Abstract

Only a minority of prostate cancers lead to death. Because no tissue biomarkers of aggressiveness other than Gleason score are available at diagnosis, many nonlethal cancers are treated aggressively. We evaluated whether a panel of biomarkers, associated with a range of disease outcomes in previous studies, could predict death from prostate cancer for men with localized disease. Using a case-only design, subjects were identified from three Australian epidemiological studies. Men who had died of their disease, “cases” (*N* = 83), were matched to “referents” (*N* = 232), those who had not died of prostate cancer, using incidence density sampling. Diagnostic tissue was retrieved to assess expression of AZGP1, MUC1, NKX3.1, p53, and PTEN by semiquantitative immunohistochemistry (IHC). Poisson regression was used to estimate mortality rate ratios (MRRs) adjusted for age, Gleason score, and stage and to estimate survival probabilities. Expression of MUC1 and p53 was associated with increased mortality (MRR 2.51, 95% CI 1.14–5.54, *P* = 0.02 and 3.08, 95% CI 1.41–6.95, *P* = 0.005, respectively), whereas AZGP1 expression was associated with decreased mortality (MRR 0.44, 95% CI 0.20–0.96, *P* = 0.04). Analyzing all markers under a combined model indicated that the three markers were independent predictors of prostate cancer death and survival. For men with localized disease at diagnosis, assessment of AZGP1, MUC1, and p53 expression in diagnostic tissue by IHC could potentially improve estimates of risk of dying from prostate cancer based only on Gleason score and clinical stage.

## Introduction

In developed countries, prostate cancer (PCa) is the most common noncutaneous cancer in males, but with the widespread use of prostate-specific antigen (PSA) testing and prostate biopsies, the majority of prostate tumors now diagnosed are localized (i.e., confined within the prostate gland), slow growing, and unlikely to metastasise 1. Despite this, and the fact that 5-year survival is generally higher than 85% 2,3, a large number of patients still opt for surgery (radical prostatectomy) due to a reduction in disease-specific mortality, especially in men younger than 65 years 4, or radiotherapy. One of the major factors driving the choice for invasive therapy is that although several prognostic algorithms have been developed based on the clinical features of a tumor, the ability of these models to predict disease outcome, and specifically prostate cancer-specific mortality (PCSM), is still quite limited 5–9. To more accurately predict which tumors are likely to remain indolent and which will progress, additional prognostic variables, such as tissue biomarkers, need to be identified and incorporated into current algorithms.

Numerous immunohistochemistry (IHC) studies have examined the prognostic value of biomarkers in PCa tumor tissue. While the majority of studies have involved small numbers of samples resulting in detected associations not holding up to multivariable analysis, recently there have been some promising results from a few larger, more robust studies. In an IHC analysis of tissue from 5718 radical prostatectomy specimens, Tsourlakis et al. found expression of the death-domain-associated protein (DAXX) to be independently associated with biochemical recurrence and to be a potential independent prognosticator of PCa outcomes 10. In two other large studies of 1826 and 7964 radical prostatectomy specimens, the NF-*κ*B and KPNA2 proteins were also found to be associated with biochemical recurrence, independently of clinical prognostic factors 11,12.

As disease prognosis and treatment decisions are the principal challenges for those patients with clinically localized disease at diagnosis 13,14, it is important to identify biomarkers that are prognostic for disease outcomes in this specific group of patients. Studying this demographic of patients, however, is in itself challenging due to the rarity of diagnostic specimen collections with outcomes data such as metastasis and PCSM. Despite these challenges, promising diagnostic biomarkers have been identified. The p16 protein was observed to be independently associated with PCSM in a multivariate analysis of tissue microarrays containing clinically localized trans-urethral resection of the prostate (TURP) specimens 15, while loss of PTEN expression in localized diagnostic specimens was significantly associated with PCSM in univariate analyses 16. Interestingly, in this latter study the association only remained significant in multivariate analyses of patients diagnosed with clinically low-risk disease and not with clinically high-risk disease. In a study of locally advanced PCa cases, p53 was observed to be significantly associated with metastasis and PCSM 17; while in a more recent, independent study of clinically localized PCa cases, Kudahetti et al. also observed an association of p53 expression with PCSM 18.

Despite these promising results, few biomarkers have been tested and validated in diagnostic tissue from localized tumors and no biomarkers have yet been established for widespread clinical usage. The aim of this study was to assess expression of a panel of tissue biomarkers including AZGP, MUC1, p53, NKX3.1, and PTEN using IHC on archival tissue collected at the time of diagnosis and to determine whether expression of these biomarkers predicts PCSM independently of Gleason score and clinical stage.

## Materials and Methods

### Sample selection

Study participants were men diagnosed with histologically confirmed PCa recruited into three epidemiological studies run by the Prostate Cancer Research Program of the Cancer Council Victoria. These studies include 964 men from the Melbourne Collaborative Cohort Study (MCCS), 786 men from the Melbourne arm of the Risk Factors for Prostate Cancer case–control study (RFPCS), and 1230 men from the Early-Onset Prostate Cancer Family Study (EOPCFS). The MCCS series included all men diagnosed with PCa during follow-up of a cohort of 41,514 volunteers (17,045 men) recruited in Melbourne during 1990–1994 19,20. For this study, follow-up ended on the 1 January 2005, which was the latest date for which complete cause of death information was available at the time of sample selection. The RFPCS is a population-based, case–control study that between 1994 and 1997 recruited men resident in Sydney, Perth, and Melbourne diagnosed with PCa at age <70 years 21,22. Eligible men with PCa were ascertained through Australian state Cancer Registries and had to be histologically confirmed with a Gleason score higher than 4. The EOPCFS is a population-based study with the principal aim of identifying genetic risk factors for early-onset PCa 23; men were recruited who had been diagnosed with PCa before age 60 years and who were reported to the Victorian Cancer Registry between 2000 and 2009. Participants in these three studies were followed passively from date of PCa diagnosis via linkage to the Victorian Registry of Births, Deaths, and Marriages (VRBDM), and the National Death Index (NDI), both of which obtain cause of death data from the Australian Bureau of Statistics.

In 2007, we began a nested case–referent study within the MCCS, RFPCS, and EOPCFS to identify factors associated with PCSM. Cases were defined as those men diagnosed with localized disease (T1–T3b) and whose death was ascribed to PCa by the Australian Bureau of Statistics (cause of death codes C61 or 185) between diagnosis and 1 January 2005 for MCCS participants, or 1 January 2007 for RFPCS and EOPCFS participants. Cause of death was reviewed by an expert coder and PCSM was confirmed for 213 men (hereafter referred as “cases”). For each case, we then randomly selected two men (hereafter referred to as “referents”) using risk set (i.e., density) sampling with years since diagnosis as the time scale. Thus, to be eligible as a referent, each man had to have survived at least as long as the corresponding case. Cases and referents were matched for Gleason score at diagnosis as recorded by the Victorian Cancer Registry (<7, 7 [3+4], 7 [4+3], 8–10, or unknown).

This study was approved by the Human Research and Ethics Committee of the Cancer Council Victoria.

### Immunohistochemical staining

Three micron tissue sections were taken from selected blocks following review and stored at 4°C in desiccated containers until used. IHC was performed using the Leica Bond-Max® autostainer (Leica Microsystems Pty Ltd, North Ryde, NSW, Australia). All primary antibody incubations were for 30 min. The detection system used was the Novocastra Novolink Max polymer (RE7320-K; Leica Microsystems Pty Ltd) with the DAKO DAB chromogen (Dako Australia Pty. Ltd., North Sydney, NSW, Australia). The antibodies used were the following: Novocastra NCL-PTEN HIER citrate 1/400 (PTEN), Novocastra NCL-MUC-1 HIER citrate 1/400 (MUC1), Novocastra-P53-D07 HIER 1/300 (p53), Zymed 35-9700 HIER citrate 1/250 (NKX3.1; Life Technologies Australia Pty Ltd, Mulgrave, Vic., Australia), and Santa Cruz SC-11358 (AZGP1 or ZAG H-123; ThermoFisher Scientific, Scoresby, Vic., Australia). Level of staining was assessed by one of the authors (J. P.), an anatomical pathologist specializing in urological cancers, who was masked to the status of the participant (i.e., case or referent). For each case the tissue block corresponding with the highest Gleason score was selected, sections were cut, and the resulting slides were stained for the five biomarkers by IHC. Biomarker expression was recorded as both the proportion of stained tumor cells (0–100%) and overall staining intensity (none, weak, moderate, or strong, i.e., 0–3+). If different Gleason patterns were present on the same slide, expression was recorded for each of them. If multiple areas with the same Gleason pattern were present on the slide, expression was averaged across them. For MUC1, AZGP1, and PTEN staining was only present in the cytoplasm while for p53 it was present only in the nucleus. For NKX3.1 we considered only cytoplasmic staining as positive as nuclear staining was ubiquitous. The data used for this study were binary, including only the proportion of stained tumor cells for the highest Gleason pattern categorized by either not expressed (<5% staining) versus expressed (≥5% staining).

Reproducibility of the IHC scoring was assessed using 20 slides for each marker which were independently assessed by another pathologist (A. L.) masked to J. P.'s results. Slides for this study were selected at random and in a way to ensure an equal distribution of expression and nonexpression of each biomarker.

### Statistical analysis

Mortality rate ratios (MRR) and 95% confidence intervals (CI) for IHC biomarker expression were estimated using Poisson regression models with PCSM as the outcome 24. For cases, duration of follow-up was calculated as the time from diagnosis to death from PCa. For referents, it was set to the value of their corresponding matched case. Restricted cubic splines with three knots (placed at the 25th, 50th, and 75th percentiles of observed log follow-up time) were used to model the baseline mortality rate. In order to account for the sampling scheme, the Poisson likelihood was weighted for each observation by the inverse probability of inclusion in the sample. Each case, therefore, had an inverse probability of inclusion of 1, and each referent had an inverse probability weight equal to half the size of the risk set from which it was sampled. This weighting scheme thus recovers the complete person-time experience of all cases eligible to be included in the case–referent study, and yields a Poisson pseudo-likelihood analogous to that of the parametric survival models described by Kalbfleisch and Lawless 24. We fitted separate models for each of AZGP1, MUC1, p53, PTEN, and NKX 3.1, as well as a combined model including all markers associated with PCSM. We estimated marginal 5-year survival probabilities using the Kaplan–Meier method for all cases and separately by Gleason score. Predicted marginal 5-year survival probabilities were calculated from the combined model for every combination of marker expression. Confidence intervals for the predicted survival function *S*(*t*) were calculated using the delta method estimate of the variance of log(−log(*S*(*t*))) back transformed to the natural metric. All models were adjusted for tumor stage, Gleason score (<7, 7 [3+4], 7 [4+3], 8–10), and age at diagnosis. The Wald test was used to compute *P*-values for coefficients.

In secondary analyses, we restricted the models to cases and referents that had been treated by radical prostatectomy. For sensitivity analyses, we fitted conditional logistic regression for the complete case–referent sets (i.e., sets including one case and at least one referent) to compare the odds ratios with the MRR obtained from the Poisson models (unconditional analysis). For the analyses conducted to determine whether the scoring of the staining was reproducible, we calculated separately for each marker the proportion of slides that were scored consistently by the two pathologists and estimated the Cohen's kappa statistics using expression in two categories (expressed vs. not expressed) as for the other analyses.

All statistical analyses were performed using Stata 12.1 for Linux 64-bit (Stata Corporation, College Station, TX).

## Results

Due to financial limitations, the present study was restricted to a randomly selected subset of 102 cases and 254 referents for whom we attempted to retrieve diagnostic archival tissue. In most instances diagnostic tissue was in the form of “cores” obtained by a trans-rectal, ultrasound-guided (TRUS) needle biopsy, in the others it was in the form of “chips” obtained by a TURP. Diagnostic tissue specimens were not able to be retrieved for 19 cases and 22 referents, leaving a total of 83 cases and 232 referents available for analysis. This sample of cases and referents was similar to the remaining 283 cases and referents for whom we did not attempt tissue collection in terms of age at diagnosis (median 64, range 41–77 years and 65, 39–79 years, respectively) and time to death (median 4.5, range 0.1–11.1 years and 5.5, 1.4–9.0 years, respectively). The distribution of Gleason score did not differ dramatically between the two groups: of those not included in this study, 30% had Gleason score less than 7, 30% had Gleason score of 7, and 40% had Gleason score of 8–10 while the proportions were 18%, 42%, and 42%, respectively for those included in this study.

The main clinical characteristics of subjects in this study are shown in Table 1. For cases, the median time between diagnosis and PCSM was 4.4 years (interquartile range 2.8–6.4 years). Analysis of the individual protein expression data revealed that those men with diagnostic tissue expressing AZGP1 had a 56% lower rate of PCSM than those with biopsies not expressing this biomarker, while expression of MUC1 or p53 was associated with a substantially increased rate of PCSM (Table 2). Associations remained remarkably strong in a combined model that included AZGP1, MUC1, and p53. Adjusting for subsequent radical prostatectomy did not materially change the estimates from the combined Poisson model, with MRRs (95% CIs) of 0.48 (0.20–1.16), 2.44 (1.03–5.75), and 3.04 (1.31–7.09) for AZGP1, MUC1, and p53, respectively. Further adjustment of the models by year of diagnosis also did not materially change the estimated MRRs.

**Table 1:** Personal and tumor characteristics of cases and referents

	Referents (*N* = 232)^1^	Cases (*N* = 83)^2^	Total (*N* = 315)
Diagnosis age, years, median (interquartile range)	63 (57–68)	64 (58–68)	63 (58–68)
Time to death, years, median (interquartile range)		4.4 (2.8–6.4)	
Diagnosis year, *N* (%)			
1992–1995	88 (38)	35 (42)	123 (39)
1996–2000	121 (52)	40 (48)	161 (51)
2001–2005	23 (10)	8 (10)	31 (10)
Gleason score^3^, *N* (%)			
<7	53 (25)	8 (11)	61 (21)
7 (3 + 4)	45 (21)	20 (26)	65 (23)
7 (4 + 3)	28 (13)	15 (20)	43 (15)
8–10	86 (41)	33 (43)	119 (41)
Clinical stage^4^, *N* (%)			
1A, 1B	60 (26)	25 (30)	85 (27)
1C	118 (52)	56 (67)	174 (56)
2B, 3A, or 3B	49 (22)	2 (2)	51 (16)

1 Referents were men with prostate cancer who were at risk of PCSM at the time of death of their corresponding case.

2 Cases were men who died during the follow-up period whose death was attributed to prostate cancer.

3 Gleason score was not available for 27 men (7 cases and 20 referents).

4 Clinical stage was unavailable for five referents.

**Table 2:** Cause-specific mortality in relation to biomarker expression^1^

	*N* referents (% expressing)^2^	*N* cases (% expressing)^3^	MRR	95% CI	*P*
Individual marker models^4^					
AZGP1 expression	219 (42%)	81 (32%)	0.44	(0.20, 0.96)	0.04
MUC1 expression	224 (64%)	82 (82%)	2.51	(1.14, 5.54)	0.02
NKX 3.1 expression	200 (70%)	78 (72%)	1.15	(0.42, 3.17)	0.79
p53 expression	219 (57%)	82 (76%)	3.08	(1.41, 6.95)	0.005
PTEN expression	221 (96%)	81 (96%)	1.11	(0.18, 6.95)	0.91
Combined model^4^					
AZGP1 expression	218 (43%)	81 (32%)	0.47	(0.20, 1.08)	0.08
MUC1 expression	218 (63%)	81 (81%)	2.10	(0.94, 4.69)	0.07
p53 expression	218 (56%)	81 (75%)	2.76	(1.23, 6.20)	0.01

1 MRR, mortality rate ratio estimated from Poisson regression models with prostate cancer-specific death as the outcome; CI, confidence interval.

2 Referents were men with prostate cancer who were at risk of prostate cancer-specific mortality at the time of death of their corresponding case.

3 Cases were men who died during the follow-up period whose death was attributed to prostate cancer.

4 Models were adjusted for age at diagnosis, stage, Gleason score, and study. Slides marked as inappropriate for data analysis by the pathologist (J. P.) were excluded. For the “individual marker models,” five different models were fitted each with only one marker included. For the “combined model,” a single model was fitted including all the three markers that had a “statistically significant” MRR from the individual marker models.

As a sensitivity analysis we also fitted a combined conditional logistic regression model using data from the 57 matched case–referent sets with at least one matched referent (i.e., a total of 57 cases and 89 referents). These results were broadly consistent with those of the combined Poisson model, with odds ratios (95% CIs) of 0.58 (0.25–1.35), 2.68 (0.94–7.59), and 1.96 (0.80–4.82) for AZGP1, MUC1, and p53 expression, respectively. A sensitivity analysis including only TRUS samples also provided similar results, with MRRs (95% CIs) of 0.35 (0.12–1.01), 1.79 (0.61–5.28), and 3.10 (1.24–7.74) for AZGP1, MUC1, and p53, respectively.

**Figure 1:**
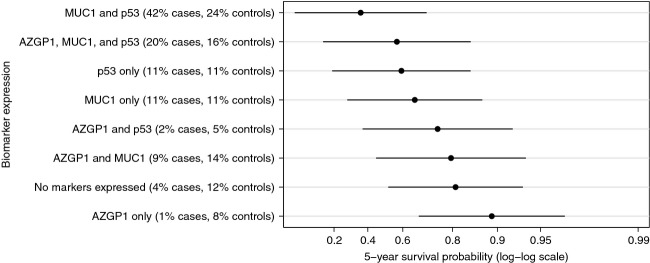
Predicted 5-year survival probabilities and 95% confidence intervals for given biomarker expression patterns. Predictions are based on the median age at diagnosis (63 years) and the most common Gleason score and clinical stage in the sample of cases (8–10 and 1c, respectively). Overall, the 5-year survival probability was 0.98 (95% CI: 0.97–0.98), and 0.84 (95% CI: 0.75–0.90) for Gleason score 8–10 prostate cancer.

**Figure 2:**
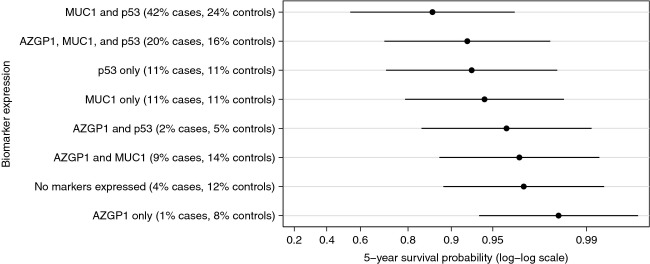
Predicted 5-year survival probabilities and 95% confidence intervals for given biomarker expression patterns. Predictions are based on the median age at diagnosis (63 years), Gleason score 6, and clinical stage 1c. Overall, the 5-year survival probability was 0.98 (95% CI: 0.97–0.98), and 0.99 (95% CI: 0.98–1.00) for Gleason score 6 prostate cancer.

We found no evidence of statistical interaction between any of the assessed markers (data not shown) and, therefore, calculated predicted marginal survival probabilities for given marker expression patterns directly from the combined Poisson model including AZGP1, MUC1, and p53. In our study the overall 5-year survival probability for men with PCa was 97% (99% for Gleason score 6 prostate cancer). The predicted 5-year disease-specific survival probabilities for a man diagnosed at the median diagnosis age (63 years), with a Gleason score 8–10 and a clinical stage T1c tumor are shown in Figure1 for each combination of marker expression. We observed substantial variation in the 5-year survival probabilities across different marker expression categories ranging from 0.90 (95% CI 0.61–0.97) for tumors expressing only AZGP1 to 0.36 (95% CI 0.05–0.71) for tumors expressing MUC1 and p53 but not AZGP1. For men with Gleason score 6 and clinical stage T1c tumors, 5-year survival probability was higher than 0.98 for tumors expressing AZGP1 only or AZGP1 and MUC-1, while it was 0.86 (95% CI 0.54–0.97) for tumors expressing MUC1 and p53 but not AZGP1 (Fig.2). The agreement in the IHC scoring of the tissue specimens between the two independent pathologists was very good for AZGP1, MUC1, p53, and PTEN with 89%, 84%, 95%, and 100% of the slides, respectively, being concordant and the *κ*-statistics being 0.76, 0.69, 0.89, and 1, respectively. For NKX3.1, the agreement was slightly lower with 78% concordant and *κ* statistics equal to 0.49.

## Discussion

Our study shows that semiquantitative IHC assessment of the expression of three proteins in diagnostic tissue, AZGP1, MUC1, and p53, taken together as a biomarker panel, predicts risk of PCSM beyond the risk predicted by “conventional” variables such as Gleason pattern and stage. Expression of AZGP1 alone was associated with a threefold decreased risk of PCSM compared with nonexpression, while MUC1 or p53 expression was associated with a twofold increased risk compared with nonexpression. Most importantly, a combined assessment of these three markers suggests that they are independent predictors of PCSM and can discriminate between cases at high risk from those at low risk of dying from PCa.

The expression of p53 protein has previously been shown to be associated with PCSM in diagnostic tumor specimens 17,18. In a series of 777 biopsy specimens from locally advanced PCa cases, Che et al. found p53 to be significantly associated with metastasis and PCSM in both univariate and multivariate analyses 17. Interestingly, when cases were stratified by treatment type, the association with PCSM only remained significant in those patients who received short-term androgen deprivation therapy with radiation therapy and not in patients who received long-term androgen deprivation therapy with radiation therapy. While our study did not have full treatment data available for stratified analyses, adjusting for subsequent radical prostatectomy did not materially change the association between p53 expression and PCSM. In second independent study of 705 TURP specimens comprising clinically localized cases, Kudahetti et al. also observed a significant association between p53 expression and PCSM 18. When cases were stratified according to Gleason score, the association only remained significant in cases with Gleason scores 8–10. We observed the expected variation in 5-year survival probabilities when looking at marker expression in different Gleason score categories. A low survival probability was observed in Gleason score 8–10 patients expressing p53 and MUC1, but not AZGP1 (0.36), whereas a higher survival probability was observed in Gleason score 6 (0.86) patients.

AZGP1 and MUC1 were first identified as candidate markers of PCa aggressiveness by a study that used cDNA microarrays to compare gene expression in fresh-frozen radical prostatectomy specimens, fresh-frozen “normal” prostate tissue from these same surgical specimens, and lymph node metastases 25. These findings were then confirmed using semiquantitative IHC on tissue microarrays of independent radical prostatectomy tumor specimens, using biochemical recurrence as outcome. MUC1 expression was shown to be higher in tumor than in normal tissue, and one study found that expression was highest in Gleason score 8–10 tumors and in lymph node metastases, although these observations were limited to a small sample 26. Another study showed that MUC1 expression correlated with increased microvessel density, a feature that is common in high-grade tumors 27. For AZGP1 the initial findings of Lapointe et al. were confirmed in a study using IHC on tissue from radical prostatectomy specimens, with clinical recurrence (i.e., localized recurrence, metastasis, or PCSM) as the outcome 28. Overall these studies provided strong evidence that expression of MUC1 and absent or weak expression of AZGP1 in radical prostatectomy specimens or tissue microarrays were associated with worse disease outcomes. Our study extends these findings and shows that expression of these two markers in diagnostic tissue is associated with PCSM that they are independent of other established predictors of disease outcome, and together with p53, allow good discrimination between men at low and high risk of PCSM.

Deletions of the tumor suppressor gene PTEN and of the transcription factor NKX3.1 are common in PCa but evidence that expression of these proteins is associated with prognosis is limited and inconclusive 16,29–32. A recent study looking at PTEN expression in 675 TURP specimens from clinically localized tumors observed that loss of PTEN expression was highly predictive of PCSM in low-risk patients (low Gleason score, low PSA, low Ki-67, or low extent of disease) but not in high-risk patients 16. Our study does not support the hypothesis that assessing the expression of these PTEN and NKX3.1 using IHC analysis on diagnostic tissue is useful for predicting disease outcome. However, it must be noted that our data are not the last word on PTEN as the MRR confidence interval for this biomarker was very wide due to ubiquitous expression in cases and referents and further investigation of this protein is warranted.

Our study has several major strengths (1) the relatively large number of cases that died from PCa and therefore, the ability to use PCSM as the outcome; (2) the simultaneous evaluation and assessment of multiple biomarkers; (3) limiting the analysis to patients with localized PCa; (4) assessing formalin-fixed, paraffin-embedded diagnostic tissue, thereby expanding the data to all patients with prostate cancer, not just those who have had radical prostatectomies or those where fresh-frozen tissue is available; (5) assessment of all biomarkers by a single, masked, expert uropathologist, and review by another, general pathologist showing that these markers could be assessed consistently; and (6) our results can be rapidly translated into practice due to the use of standard prostate diagnostic tissue (formalin-fixed and paraffin-embedded) and IHC procedures using commercially available antibodies. We selected IHC to assess protein expression as it is used by virtually all pathologists, including those in middle- or low-income countries and because it is simple and inexpensive (and where applicable, already reimbursed by government and private insurers).

Despite the many strengths mentioned above, our study does have some limitations. First, serum PSA concentrations at diagnosis were not available for our patients, although this omission is unlikely to have confounded the observed associations. PSA at diagnosis is a strong predictor of biochemical relapse 6 but not of PCSM, of which the strongest predictor is Gleason score 7. Another potential limitation is that radical prostatectomy was the only treatment information available and previous studies have noted that biomarker prognostication does vary between treatment groups 17. However, it should be noted that the associations between marker expression and PCSM were virtually unchanged when estimated for the subset of surgically treated patients, suggesting that treatment may not materially influence our results. The matching of cases and referents was based on Gleason scores from the original diagnostic histopathology reports, and these same Gleason scores were used in our analyses. It is possible that the grading of prostate tumors has changed over the recruitment period of the three studies (1990–2009), but as this would affect both cases and referents equally it is unlikely to have influenced our estimates of association. This assertion is corroborated by the fact that adjustment for year at diagnosis did not materially change the MRRs (data not shown). The strong similarity between the MRRs from the Poisson models and the odds ratios from the conditional logistic regression indicates that the choice of Gleason score categories has a minimal impact, if any, on the association between expression of the tissue biomarkers and risk of PCSM. Finally, while our p53 results validate those of two previous studies, and together provide compelling evidence for the use of this biomarker in prognostication, a validation dataset was not available for our full panel of biomarkers and therefore these results need to be confirmed in an independent case series.

In summary, our results, if validated in an independent set of cases, show that a panel of markers, including AZGP1, MUC1, and p53, could be assessed using routine IHC at the time of diagnosis of clinically localized PCa. This panel may help clinicians and patients make more informed decisions around treatment options and therefore decrease unnecessary treatment for men at low risk of dying from the disease and improve our ability to identify men that might benefit from aggressive treatment.
